# Elevated non-HDL-C/HDL-C ratio increases the 1-year risk of recurrent stroke in older patients with non-disabling ischemic cerebrovascular events: results from the Xi’an Stroke Registry Study of China

**DOI:** 10.1186/s12877-023-04102-x

**Published:** 2023-07-05

**Authors:** Zhongzhong Liu, Xuemei Lin, Lingxia Zeng, Huan Zhang, Weiyan Guo, Qingli Lu, Congli Huang, Jing Wang, Pei Liu, Qiaoqiao Chang, Mi Zhang, Yan Huo, Yan Wang, Fang Wang, Songdi Wu

**Affiliations:** 1 Department of Neurology, Xi’an No.1 Hospital, The First Affiliated Hospital of Northwest University, Xi’an, 710002 China; 2 Department of Epidemiology and Biostatistics, School of Public Health of Xi’an Jiaotong University Health Science Center, Xi’an, 71006 China; 3 College of Life Science, Northwest University, Xi’an, 710069 China; 4 Xi’an Key Laboratory for Innovation and Translation of Neuroimmunological Diseases, Xi’an, 710002 China; 5 Department of Traditional Chinese Medicine, Xi’an No.1 Hospital, The First Affiliated Hospital of Northwest University, Xi’an, 710002 China

**Keywords:** Non-disabling ischemic cerebrovascular events, Lipids, Older patients, Recurrent stroke

## Abstract

**Background:**

Few studies have explored the prognostic role of nontraditional lipid-related indicators in non-disabling ischemic cerebrovascular events (NICE). In this study, we aimed to investigate the relationship between the ratio of non-high-density lipoprotein cholesterol to high-density lipoprotein cholesterol (non-HDL-C/HDL-C) and the1-year risk of recurrent stroke in patients with NICE.

**Methods:**

Total cholesterol (TC), HDL-C, and patient information were collected at admission. Recurrent stroke events were followed up 3, 6, and 12 months after onset. Non-HDL-C levels were calculated by subtracting HDL-C from TC. The non-HDL-C/HDL-C ratio was treated as a continuous variable and in quartiles (Q1–Q4). Stratified multivariate Cox regression was used to investigate the relationship between the non-HDL-C/HDL-C ratio and the 1-year risk of recurrent stroke in patients with NICE.

**Results:**

Overall, 1,659 patients with NICE were enrolled. For each unit increase in the non-HDL-C/HDL-C ratio, the 1-year risk of recurrent stroke in patients aged ≥ 65 years (older patients) with NICE increased by 64% in the adjusted model (hazard ratio [HR]: 1.64, 95%confidence interval [CI]:1.18–2.27, *P* = 0.003), and the HRs were 3.21 and 4.24 times higher in the Q3 and Q4 groups than that in the Q1 group, which was considered to be the reference (adjusted model Q3: HR: 3.21, 95%CI: 1.05–9.83, *P* = 0.041; adjusted model Q4: HR: 4.24, 95%CI: 1.30–13.85, *P* = 0.017). However, there was no significant difference in patients younger than 65 years. Both curve fitting and Kaplan–Meier cumulative risk analysis showed that an elevated non-HDL-C/HDL-C ratio significantly increased the 1-year risk of recurrent stroke in older patients with NICE. The optimal range for the non-HDL-C/HDL-C ratio should be no higher than the Q2 group (2.256–2.939). Stratified Cox regression analysis showed that these results tended to be stable for different comorbidities (all *P* for interaction > 0.05).

**Conclusions:**

Elevated non-HDL-C/HDL-C ratios significantly increased the 1-year risk of recurrent stroke in older patients with NICE. Therefore, clinicians need to pay more attention to this indicator when managing older patients with NICE.

**Supplementary Information:**

The online version contains supplementary material available at 10.1186/s12877-023-04102-x.

## Background

Non-disabling ischemic cerebrovascular events (NICE), including transient ischemic attack (TIA), minor ischemic stroke, rapid remission, and unimpaired stroke, have attracted significant attention in recent years [1]. The incidence rate of recurrent stroke and cardiovascular events in the first year in patients with NICE is > 6% [1, 2]. Patients with NICE are more likely to be treated aggressively and intensively than those with disabling ischemic cerebrovascular events, thus attracting clinical attention [3, 4].

Previous studies have shown that nontraditional serum lipid-related indicators (including total cholesterol [TC]/high-density lipoprotein cholesterol [HDL-C] ratio, triglycerides [TGs]/HDL-C ratio, and non-HDL-C) are associated with coronary heart disease, asymptomatic intracranial arterial stenosis, and stroke prognosis [5–7]. Non-HDL-C was obtained as a rough measure of TC minus HDL-C and represented the cholesterol content in the atherogenic lipoproteins [3, 8]. The non-HDL-C/HDL-C ratio is obtained by dividing the non-HDL-C levels by the HDL-C levels. This ratio is a nontraditional indicator of dyslipidemia, representing the balance between atherogenic and antiatherogenic lipid particles. It is significantly associated with an increased prevalence of asymptomatic intracranial arterial stenosis [7] and carotid atherosclerosis [9]. In addition, the non-HDL-C/HDL-C ratio is strongly associated with several dyslipidemia-related diseases, such as diabetes mellitus [10], liver function test abnormalities [11], chronic kidney disease [12], and carotid atherosclerosis [9] as well as considered superior to traditional lipid profiles in assessing the degree of arterial stiffness [13].

Many studies have reported that the non-HDL-C/HDL-C ratio is associated with carotid atherosclerosis, intracranial atherosclerotic stenosis, and poor stroke prognosis [5, 14–16]. However, few studies have focused on the relationship between the non-HDL-C/HDL-C ratio and the 1-year risk of recurrent stroke in patients with NICE who have a high incidence of recurrent stroke [2, 17]. Therefore, we hypothesized that the non-HDL-C/HDL-C ratio might have a predictive value for recurrent stroke within 1 year in patients with NICE.

Based on the Xi’an Stroke Registry Study platform in China, we collected TC, HDL-C, and other clinical data to investigate the relationship between the non-HDL-C/HDL-C ratio and the 1-year risk of recurrent stroke in patients with NICE. We also aimed to explore new cholesterol intervention targets for reducing the 1-year risk of recurrent stroke in patients with NICE.

## Methods

### Study population

**Fig. 1:**
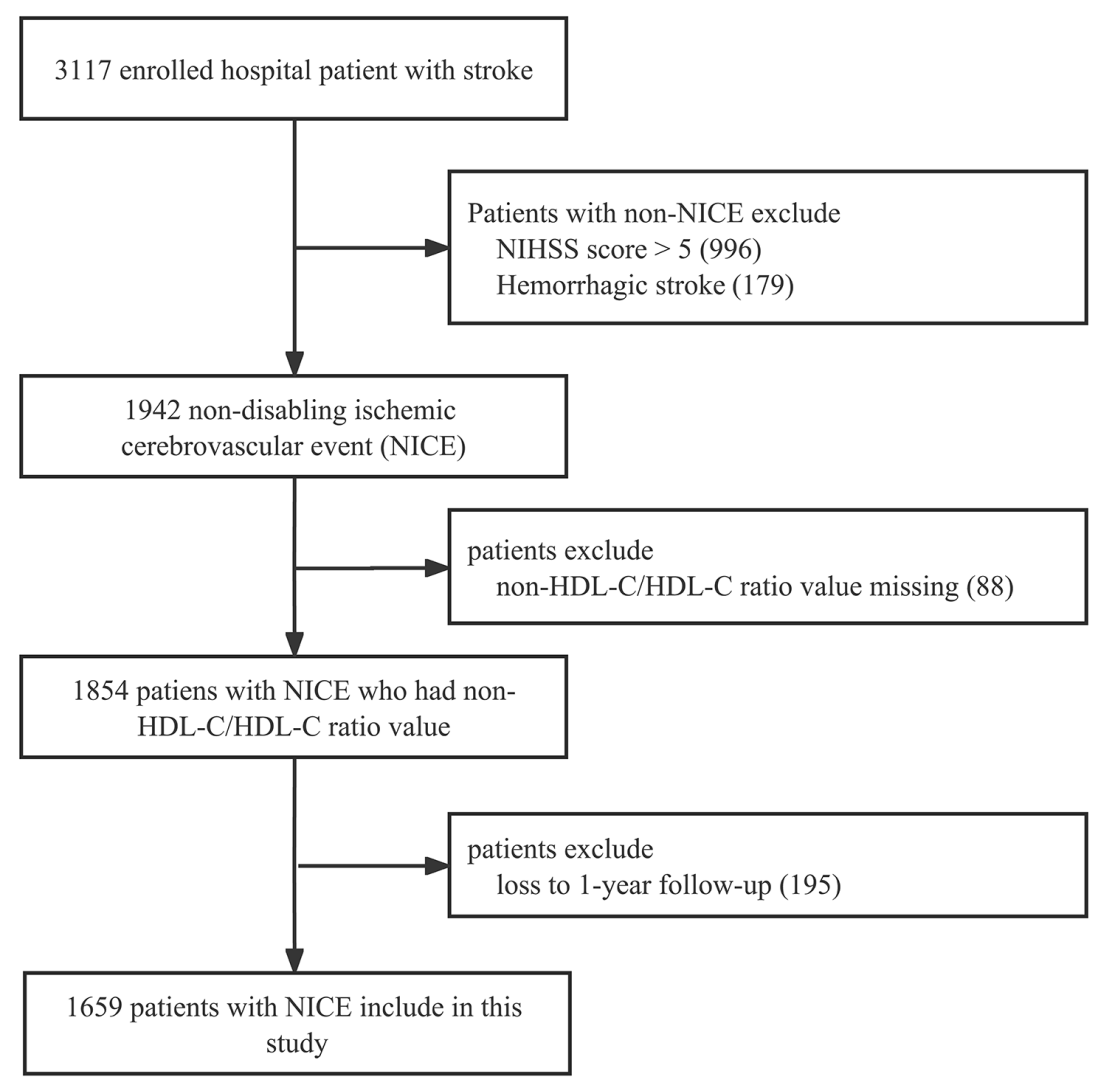
Flow chart of the screening and enrollment of study participants. NICE, non-disabling ischemic cerebrovascular events; NIHSS, National Institutes of Health Stroke Scale; HDL-C, high-density lipoprotein cholesterol

This study is based on 3,117 patients with all stroke subtypes collected in the Xi’an Stroke Registry Study platform from January to December 2015 [18]. These patients were hospitalized for stroke and were followed up at 3, 6, and 12 months after onset. We excluded non-NICE patients, including those with a NIHSS score on admission > 5 (n = 996), and those with hemorrhagic stroke (n = 179). In addition, we also excluded patients who do not have data on non-HDL-C/HDL-C ratio (n = 88), and patients who failed to follow-up at the end of 1 year (n = 195). Finally, a total of 1659 patients with NICE were included. Detailed inclusion and exclusion criteria are presented in Fig. 1. NICE includes minor ischemic stroke, TIA, rapid remission, and unimpaired stroke. Minor ischemic stroke was defined as an NIHSS score on admission ≤ 5 [19]. The TIA was defined as focal brain ischemia with resolution of symptoms within 24 h after onset, which is consistent with the definition used in Wang et al.‘s study [4]. Rapid remission and unimpaired stroke were defined as NIHSS > 5 at the onset & NIHSS 0–5 at arrival. The diagnostic criteria were consistent across all the participating hospitals. Follow-up was conducted from January 2015 to February 2017.

This study conforms to the guiding principles of the Declaration of Helsinki. It has been approved by the academic committee of Xi’an No.1 Hospital and the ethics committees of each participating hospital (approval no. 2014 [5]). Written informed consent was obtained from all patients.

### Measurements and outcomes

This was a prospective, multicenter, observational cohort study. We obtained baseline data, including demographic information (age, sex, and educational level), cerebrovascular risk factors (including smoking and drinking), and examination on admission (including body mass index [BMI], systolic blood pressure [SBP] on admission, diastolic blood pressure [DBP]on admission, heart rate, NIHSS score on admission). We also obtained previous medical history (including prior stroke, hypertension, diabetes mellitus, and atrial fibrillation) and pneumonia. We looked at laboratory data, including TC, TGs, HDL-C, low-density lipoprotein cholesterol (LDL-C), glycated hemoglobin, fasting blood glucose (FBG), alanine aminotransferase, aspartate aminotransferase, alkaline phosphatase, homocysteine, serum creatinine, estimated glomerular filtration rate (eGFR), blood urea nitrogen, uric acid, leukocyte count, and platelet count (Table 1).

**Table 1:** Baseline characteristics by non-HDL-C/HDL-C ratio quartiles (Q1-Q4) in patients with NICE.

Variables	Overall n = 1659	Non-HDL-C/HDL-C ratio quartiles				P value
		Q1, n = 415	Q2, n = 414	Q3, n = 415	Q4, n = 415	
**Demographic information**						
Age (years)	64.0 ± 12.1	66.3 ± 12.3	64.1 ± 12.3	64.1 ± 11.2	61.5 ± 12.0	< 0.001
Sex, n(%)						0.116
male	1050(63.3)	266(64.1)	247(59.7)	257(61.9)	280(67.5)	
female	609(36.7)	149(35.9)	167(40.3)	158(38.1)	135(32.5)	
Educational level, n (%)						0.03
elementary or below	756(45.6)	217(52.3)	180(43.5)	176(42.4)	183(44.1)	
middle school	302(18.2)	67(16.1)	67(16.2)	85(20.5)	83(20)	
high school or above	601(36.2)	131(31.6)	167(40.3)	154(37.1)	149(35.9)	
**Cerebrovascular risk factors**						
Smoking, n (%)						0.006
Never smoking	925(55.8)	218(52.5)	254(61.4)	236(56.9)	217(52.3)	
smoking cessation	318(19.2)	100(24.1)	69(16.7)	77(18.6)	72(17.3)	
current smoking	416(25.1)	97(23.4)	91(22.0)	102(24.6)	126(30.4)	
Drinking, n (%)	400(24.1)	95(22.9)	81(19.6)	107(25.8)	117(28.2)	0.024
**Examination on admission**						
BMI (kg/m^2^)	24.0 ± 3.3	23.3 ± 3.6	23.8 ± 3.5	24.1 ± 3.1	24.6 ± 2.9	< 0.001
SBP on admission (mmHg)	145.4 ± 20.8	144.9 ± 20.8	145.8 ± 22.5	144.2 ± 19.4	146.6 ± 20.4	0.363
DBP on admission (mmHg)	85.5 ± 12.3	84.0 ± 11.4	85.7 ± 13.2	85.1 ± 11.5	87.3 ± 12.6	0.001
Heart rate (times per minute)	74.5 ± 9.6	74.5 ± 9.8	75.0 ± 10.3	74.5 ± 8.8	73.9 ± 9.5	0.424
NIHSS score on admission	2.0(1.0 ~ 4.0)	3.0(1.0 ~ 4.0)	2.0(1.0 ~ 4.0)	2.0(1.0 ~ 4.0)	2.0(0.0 ~ 4.0)	0.226
**Previous medical history**						
Prior stroke, n (%)	442(26.6)	117(28.2)	113(27.3)	100(24.1)	112(27)	0.572
Hypertension, n (%)	1181(71.2)	285(68.7)	294(71)	292(70.4)	310(74.7)	0.271
Diabetes mellitus, n (%)	378(22.8)	65(15.7)	94(22.7)	88(21.2)	131(31.6)	< 0.001
Atrial fibrillation, n (%)	74(4.5)	20(4.8)	23(5.6)	22(5.3)	9(2.2)	0.069
**Pneumonia**, n (%)	44(2.7)	12(2.9)	8(1.9)	13(3.1)	11(2.7)	0.731
**Laboratory findings**						
Total cholesterol (mmol/L)	4.4 ± 1.1	3.7 ± 0.8	4.2 ± 0.9	4.6 ± 0.9	5.1 ± 1.1	< 0.001
Triglycerides (mmol/L)	1.7 ± 1.4	1.2 ± 0.9	1.5 ± 0.9	1.7 ± 1.0	2.5 ± 2.0	< 0.001
HDL-cholesterol (mmol/L)	1.1 ± 0.3	1.4 ± 0.3	1.2 ± 0.3	1.1 ± 0.2	0.9 ± 0.2	< 0.001
LDL-cholesterol (mmol/L)	2.6 ± 0.8	2.0 ± 0.6	2.5 ± 0.7	2.8 ± 0.7	3.1 ± 0.9	< 0.001
Glycated hemoglobin,%	6.4 ± 1.6	6.0 ± 1.3	6.2 ± 1.5	6.4 ± 1.5	6.9 ± 1.9	< 0.001
FBG (mmol/L)	5.9 ± 2.3	5.5 ± 1.8	5.7 ± 2.0	5.9 ± 2.1	6.4 ± 2.8	< 0.001
Alanine aminotransferase (U/L)	23.7 ± 18.4	23.4 ± 17.8	22.5 ± 14.1	24.4 ± 23.2	24.6 ± 17.4	0.343
Aspartate aminotransferase (U/L)	23.8 ± 12.5	24.5 ± 11.7	23.9 ± 11.7	23.7 ± 15.4	23.2 ± 10.9	0.482
Alkaline phosphatase (U/L)	78.3 ± 25.4	77.2 ± 28.4	79.8 ± 27.1	77.8 ± 22.9	78.3 ± 22.9	0.52
Homocysteine (µmol/mL)	21.1 ± 14.2	19.5 ± 12.7	20.5 ± 13.8	21.5 ± 15.1	23.0 ± 15.0	0.035
Serum creatinine (µmol/L)	75.8 ± 38.5	73.3 ± 28.6	75.0 ± 42.7	74.4 ± 22.4	80.4 ± 52.5	0.042
eGFR (mL/min/1.73m^2^)	75.8 ± 17.7	74.1 ± 19.2	75.9 ± 18.2	76.5 ± 15.1	76.7 ± 18.1	0.133
Blood urea nitrogen(mmol/L)	5.0 ± 1.8	5.2 ± 2.1	5.0 ± 1.7	5.0 ± 1.7	5.0 ± 1.7	0.481
Uric acid(µmol/L)	292.6 ± 96.5	281.9 ± 99.8	286.7 ± 95.9	294.5 ± 92.6	307.2 ± 95.9	0.001
Leukocyte count (×10^9^/L)	6.7 ± 2.3	6.4 ± 2.3	6.6 ± 2.2	6.8 ± 2.3	7.0 ± 2.3	< 0.001
Platelet count (×10^9^/L)	191.0 ± 59.2	179.7 ± 59.9	185.7 ± 57.0	197.2 ± 58.9	201.6 ± 58.6	< 0.001

Notes: data presented are mean ± SD, median (Q1–Q3), or N (%)

Abbreviations: BMI, body mass index; FBG, fasting blood glucose; NIHSS, national institutes of health stroke scale; SBP, systolic blood pressure; DBP, diastolic blood pressure; HDL, high-density lipoprotein; LDL, low-density lipoprotein; eGFR, estimated glomerular filtration rate. Q, quartilesQ1: <2.256, Q2:2.256–2.939,Q3: 2.940–3.749,Q4: ≥3.750

The definition and evaluation criteria of clinically related data in this study were the same as those in the Chinese Intracranial Atherosclerosis Study [20]. Lipid-related indicators, including TC, LDL-C, HDL-C, and TG, were measured through venous blood within 24 h after admission. Non-HDL-C was calculated by subtracting HDL-C from TC. The non-HDL-C/HDL-C ratio was obtained by dividing non-HDL-C by HDL-C levels and was treated as continuous and quartile (Q1–Q4) variables for analysis. Non-HDL-C/HDL-C ratio quartiles (Q1–Q4) were defined as the distribution of non-HDL-C/HDL-C ratio from low to high and divided into four parts according to the quartiles. The ranges of quartiles (Q1–Q4) were as follows: Q1: <2.256, Q2: 2.256–2.939, Q3: 2.940–3.749, and Q4: ≥3.750.

The endpoint event of this study was recurrent stroke within 1 year, defined as the occurrence of new acute stroke events (including acute ischemic stroke, intracranial hemorrhage, subarachnoid hemorrhage, and TIA) during the 1-year follow-up [21]. New acute stroke events were determined by an independent adjudication board composed of stroke specialists from each hospital.

### Follow-up

The patients were followed-up at 3, 6, and 12 months after stroke onset, with a time error within 7 days. Data on recurrent stroke were collected via telephone or face-to-face interviews. All follow-up processes were completed by trained research coordinators. For patients with recurrent stroke, the date of the event was recorded. Patients who withdrew from the study or could not be contacted for 5 consecutive working days were considered lost to follow-up.

### Statistical analyses

Continuous variables conforming to normal distribution are presented as mean ± standard deviation, and categorical variables are presented as percentages (%). The Chi-square or t-test was used to analyze the differences between the two groups. One-way analysis of variance was used to assess differences among multiple groups of normally distributed variables with consistent variance. Multiple continuous variables among multiple groups with incomplete normal distribution were compared using the Kruskal–Wallis rank-sum test. Multivariate Cox regression models were used to analyze the relationship between the non-HDL-C/HDL-C ratio and the 1-year risk of recurrent stroke. The covariates were included as potential confounders based on their relationship with the outcomes of interest or a change in effect estimate of more than 10%.

Smooth curve fitting and Kaplan–Meier (K–M) curves were used to analyze the nonlinear relationship between the non-HDL-C/HDL-C ratio and the 1-year risk of recurrent stroke. The log-rank test was used to detect differences in the recurrence curves among quartiles. Subgroup analysis and forest maps were used to analyze the influence of related risk factors and comorbidities on the relationship between the non-HDL-C/HDL-C ratio and the 1-year risk of recurrent stroke. All analyses were performed using the statistical software packages R 3.3.2 (http://www.R-project.org, The R Foundation) and EmpowerStats (R) (www.empowerstats.com, X&Y Solutions, Inc., Boston, MA, USA).

## Results

### Baseline characteristics

At the end of the follow-up period, 195 patients were lost to follow-up. We compared the clinical characteristics of patients lost to follow-up with those who were not lost to follow-up at the end of 1 year. We found that only SBP and DBP at admission significantly differed between the two groups, while the other related clinical characteristics showed no significant difference. Those findings suggest that the patients who were lost to follow-up were close to randomization and had little impact on the relationship between the non-HDL-C/HDL-C ratio and the 1-year risk of recurrent stroke (Supplementary Table 1).

The cohort used in our study consisted of 1659 eligible participants with a mean age of 64.0 ± 12.1 years (male: 63.3%). Baseline demographic information, cerebrovascular risk factors, examinations on admission, previous medical histories, and laboratory findings of the non-HDL-C/HDL-C ratio quartiles (Q1–Q4) were compared (Table 1). Patients with a higher non-HDL-C/HDL-C ratio tended to be relatively younger, had higher smoking and drinking rates, lower HDL-C levels, and a higher prevalence of diabetes mellitus, as well as higher education status compared to those with a lower non-HDL-C/HDL-C ratio. In addition, the non-HDL-C/HDL-C ratio was directly proportional to BMI, DBP on admission, TC, TG, LDL-C, glycated hemoglobin, FBG, homocysteine, serum creatinine, and uric acid levels, leukocyte, and platelet counts. The non-HDL-C/HDL-C ratio among quartiles showed no significant differences in terms of sex, SBP on admission, alanine aminotransferase, aspartate aminotransferase, heart rate, NIHSS score on admission, prior stroke, hypertension, atrial fibrillation, pneumonia, alkaline phosphatase, blood urea nitrogen levels, and eGFR.

### Cox regression analysis of the non-HDL-C/HDL-C ratio and 1-year risk of recurrent stroke in patients with NICE

In the crude model without adjustments, our results revealed that the Cox regression analysis did not identify a significant association between an increase in the non-HDL-C/HDL-C ratio whether considered as a continuous or categorical variable, and the 1-year risk of recurrent stroke. However, after adjusting for potential confounders in the adjusted model, the 1-year risk of recurrent stroke increased by 43% for each unit increase in the non-HDL-C/HDL-C ratio in patients with NICE (HR: 1.43, 95%CI: 1.11–1.84, *P* = 0.005). The 1-year risk of recurrent stroke was 2.70-fold higher in Q4 than in Q1, which was considered the reference in patients with NICE (HR: 2.70, 95%CI: 1.06–6.89, *P* = 0.037) (Table 2). Further analysis revealed that age, as an important confounder, significantly affected the results of the univariate and multivariate Cox regression analyses. Therefore, we performed Cox regression analysis stratified by age < 55, 55–64, 65–74, and ≥ 75 years. The results showed that the 1-year risk of recurrent stroke did not significantly increase with the non-HDL-C/HDL-C ratio in patients aged < 65 years with NICE. The risk significantly increased in ages 65–74 years (HR: 1.92, 95%CI: 1.16 ~ 3.18, *P* = 0.011) and ages ≥ 75 years (HR: 1.73, 95%CI: 1.04 ~ 2.88, *P* = 0.036) (Supplementary Table 2).

**Table 2:** Cox regression analysis of the non-HDL-C/HDL-C ratio and 1-year risk of recurrent stroke in patients with NICE

Variable	Overall	Event, n%	Crude model HR(95%CI)	*P* value	Adjusted model HR(95%CI)	*P* value
Non-HDL-C/HDL-C ratio, per 1 unit increase	1659	58(3.5)	1.09(0.89 ~ 1.33)	0.425	1.43(1.11 ~ 1.84)	0.005
Non-HDL-C/HDL-C ratio quartile						
Q1	415	11(2.7)	Ref.		Ref.	
Q2	414	15(3.6)	1.37(0.63 ~ 2.97)	0.432	1.79(0.75 ~ 4.27)	0.189
Q3	415	16(3.9)	1.45(0.68 ~ 3.13)	0.339	2.27(0.96 ~ 5.38)	0.063
Q4	415	16(3.9)	1.45(0.67 ~ 3.12)	0.344	2.70(1.06 ~ 6.89)	0.037
*P* for trend				0.355		0.03

Notes: Crude adjust none; adjusted for age, sex; smoking, drinking, prior stroke, pneumonia, NIHSS score at admission, BMI, ALT, triglyceride, FBG, alkaline phosphatase, platelet count, blood urea nitrogen, hypertension, atrial fibrillation, and diabetes mellitus. Abbreviations: BMI, body mass index; FBG, fasting blood glucose; NIHSS, national institutes of health stroke scale; ALT, alanine aminotransferase; HR, hazard ratio; CI, confidence interval; and Q, quartile. Q1: <2.256, Q2:2.256–2.939, Q3: 2.940–3.749, Q4: ≥3.750

### Analyzing the relationship between the non-HDL-C/HDL-C ratio and the 1-year risk of recurrent stroke in patients with NICE using age-stratified analysis

**Fig. 2:**
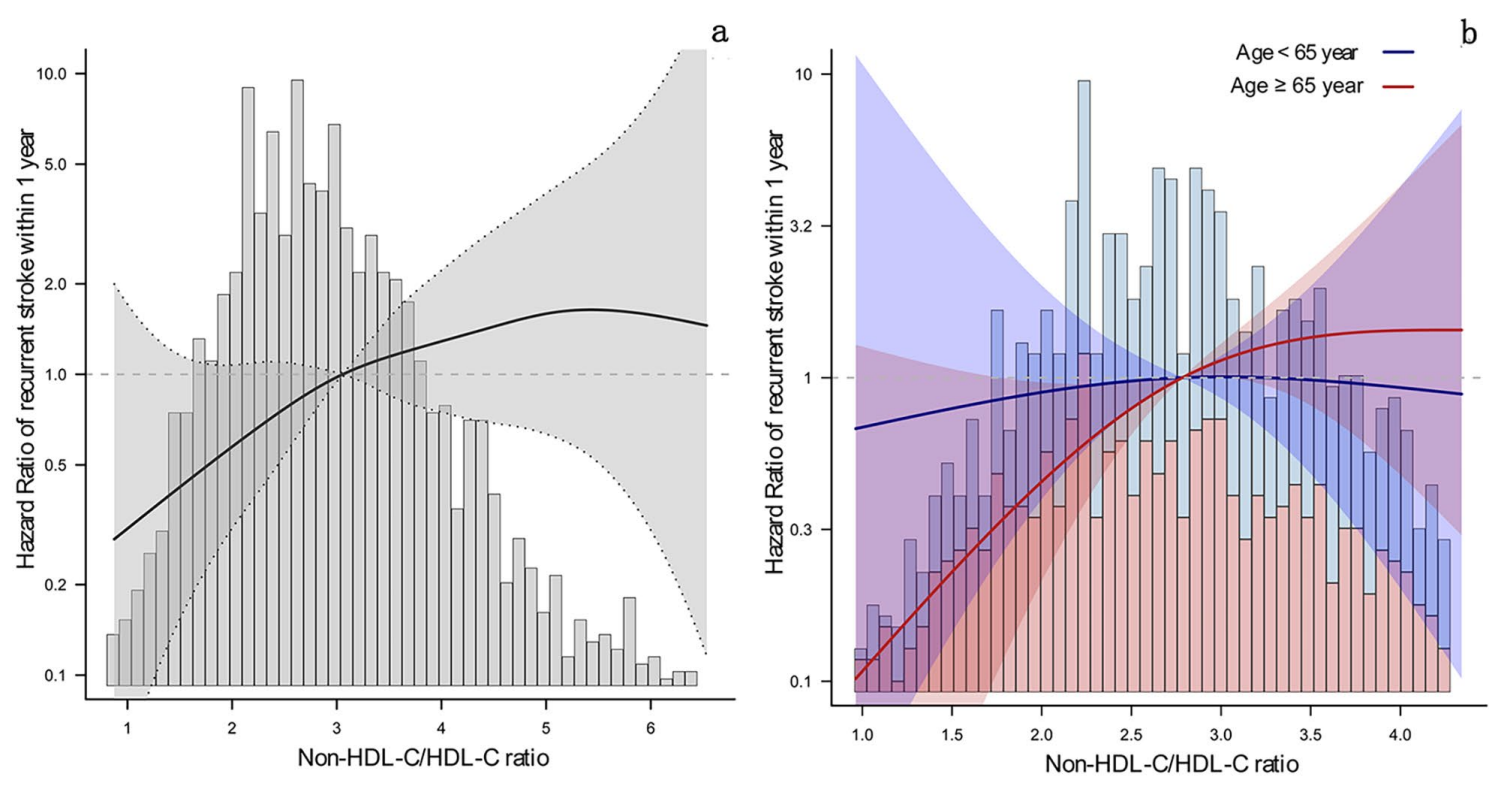
The curve fitting of non-HDL-c/HDL-c ratio and 1-year risk of recurrent stroke in patients with NICE. Figure 2**a**, represents curve fitting regardless of all ages; Fig. 2**b**, represents curve fitting grouped by age ≥ 65 years. The solid line indicates the adjusted hazard ratio and the dashed lines the 95% confidence interval bands. Age ≥ 65 year were shown in red, and age < 65 years were shown in blue

Based on the above analysis, patients with NICE were divided into two groups according to their age, i.e., patients who were aged < 65 years and those aged ≥ 65 years. The results of the Cox regression analysis are shown in Table 3. On the one hand, When treating the non-HDL-C/HDL-C ratio as a continuous variable, for each unit increase of non-HDL-C/HDL-C ratio in patients aged < 65 years with NICE, there were no significant differences in the crude and adjusted models (crude model: HR: 0.91, 95%CI: 0.63–1.32, *P* = 0.61; adjusted model: HR: 1.23, 95%CI: 0.77–1.95, *P* = 0.391). However, for older patients (≥ 65 years old) with NICE, the 1-year risk of recurrent stroke increased by 33% in the crude model and 64% in the adjusted model (crude model: HR: 1.33, 95%CI: 1.02–1.74, *P* = 0.038; adjusted model: HR: 1.64, 95%CI: 1.18–2.27, *P* = 0.003). The smoothing curve analysis showed an increased linear relationship that went up from left to right between the non-HDL-C/HDL-C ratio and the 1-year risk of recurrent stroke in all age groups of NICE patients (Fig. 2a). However, stratified curve fitting showed that this linear relationship was mainly present in older patients (Fig. 2b).

**Table 3:** Multivariate analysis of non-HDL-C/HDL-C ratio and 1-year risk of recurrent stroke in patients with NICE stratified by age of 65 years

Subgroup	Variable	Overall	Event, n%	Crude Model HR(95%CI)	*P* value	Adjusted Model HR(95%CI)	*P* value
**Age < 65 year**							
	Non-HDL-C/HDL-C ratio, per 1 unit increase	833	19(2.3)	0.91(0.63 ~ 1.32)	0.61	1.23(0.77 ~ 1.95)	0.391
	Non-HDL-C/HDL-Cratio quartile						
	Q1	178	5 (2.8)	Ref.		Ref.	
	Q2	201	6 (3)	1.08 (0.33 ~ 3.53)	0.902	1.98(0.43 ~ 9.15)	0.384
	Q3	206	4 (1.9)	0.69 (0.19 ~ 2.59)	0.587	1.40(0.26 ~ 7.53)	0.697
	Q4	248	4 (1.6)	0.57 (0.15 ~ 2.13)	0.406	1.36(0.22 ~ 8.37)	0.744
	*P* for trend				0.31		0.854
**Age≥65 year**							
	Non-HDL-C/HDL-C ratio, per 1 unit increase	826	39(4.7)	1.33(1.02 ~ 1.74)	0.038	1.64(1.18 ~ 2.27)	0.003
	Non-HDL-C/HDL-C ratio quartile						
	Q1	237	6 (2.5)	Ref.		Ref.	
	Q2	213	9 (4.2)	1.65 (0.59 ~ 4.64)	0.341	2.02(0.64 ~ 6.39)	0.229
	Q3	209	12 (5.7)	2.26 (0.85 ~ 6.03)	0.102	3.21(1.05 ~ 9.83)	0.041
	Q4	167	12 (7.2)	2.85 (1.07 ~ 7.60)	0.036	4.24(1.3 ~ 13.85)	0.017
	*P* for trend				0.025		0.011

Notes: Crude adjust none; adjusted for sex; smoking, drinking, prior stroke, pneumonia, NIHSS score at admission, BMI, ALT, triglyceride, FBG, alkaline phosphatase, platelet count, blood urea nitrogen, hypertension, atrial fibrillation, and diabetes mellitus. Abbreviations: BMI, body mass index; FBG, fasting blood glucose; NIHSS, national institutes of health stroke scale; ALT, alanine aminotransferase; HR, hazard ratio; CI, confidence interval; and Q, quartiles. Q1: <2.256, Q2:2.256–2.939, Q3: 2.940–3.749, Q4: ≥3.750

**Fig. 3:**
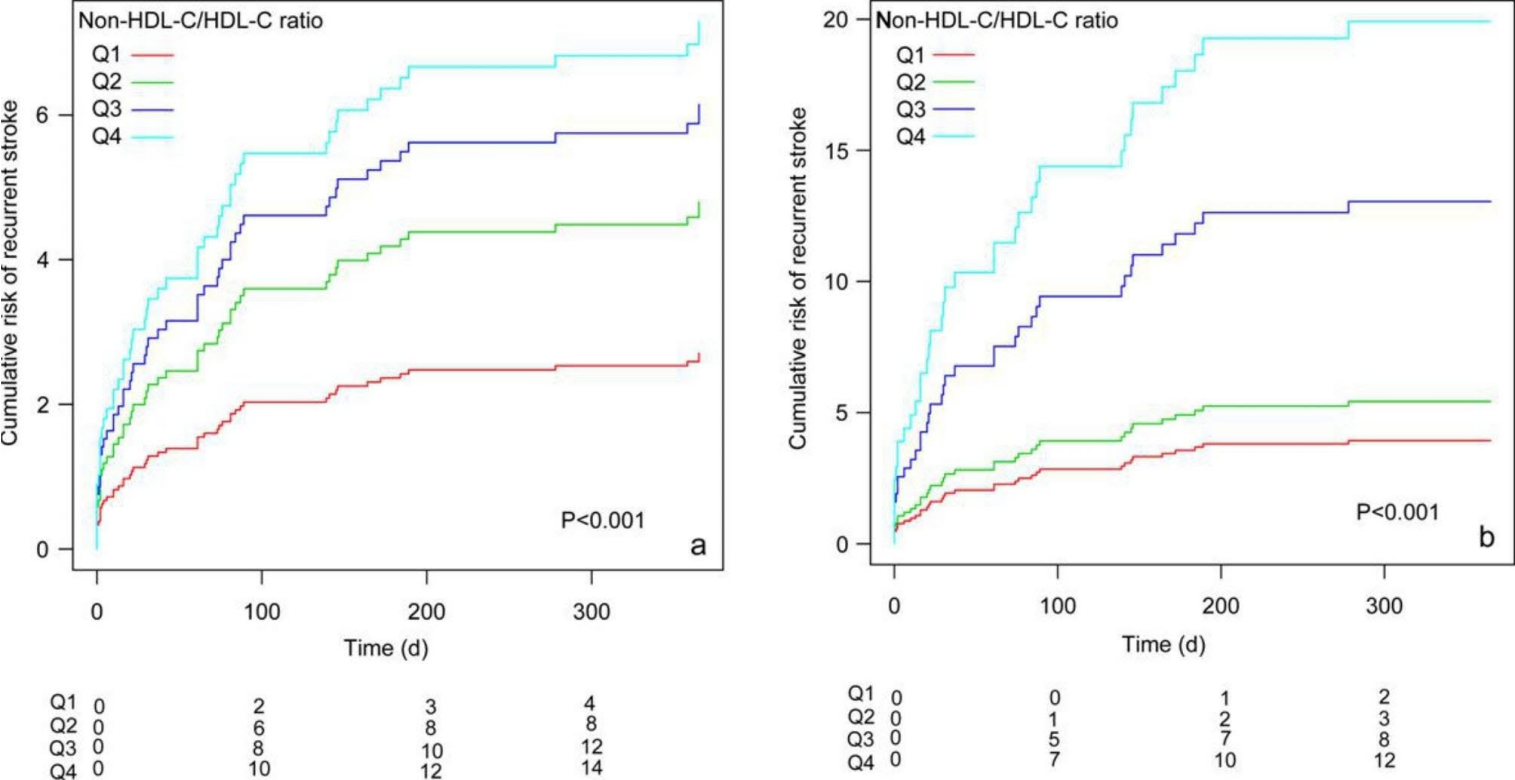
Cumulative risk of recurrent stroke within 1 year after non-HDL-C/HDL-C ratio as quartiles (Q1-Q4). Figure 3**a** represents cumulative risk regardless of all ages; Fig. 3**b** represents cumulative risk grouped by age ≥ 65 years

On the other hand, when treating the non-HDL-C /HDL-C ratio as a categorical variable (Q1-Q4). The 1-year risk of recurrent stroke in the Q4 group was 2.85 times higher than that in the Q1 group, which was considered the reference (crude model: HR: 2.85, 95%CI: 1.07–7.60, *P* = 0.036). The risks of the 1-year risk of recurrent stroke in the Q3 and Q4 groups were 3.21 and 4.24 times higher than in the Q1 group, respectively (adjusted model Q3: HR: 3.21, 95%CI: 1.05–9.83, *P* = 0.041; adjusted model Q4: HR: 4.24, 95%CI: 1.30–13.85, *P* = 0.017). Although there was no significant difference between Q2 and Q1, the trend of increased risk was significantly different in the Q1-Q4 groups(*P* = 0.025 and *P* = 0.011, respectively).In addition, after adjusting for potential confounding factors (the K-M curve of univariate analysis was illustrated in Supplementary Fig. 1), the K-M cumulative risk analyses also showed that the cumulative risk of the 1-year risk of recurrent stroke increased with an increase in the non-HDL-C/HDL-C ratio from Q1 to Q4 in all age groups (Fig. 3a). The 1-year risk of recurrent stroke was significantly higher in the Q3 and Q4 groups than in the Q1 group, mainly in older patients. The optimal range of the non-HDL-C/HDL-C ratio should be no higher than the Q2 group (2.256–2.939) (Fig. 3b).

### Subgroups analyses

**Fig. 4:**
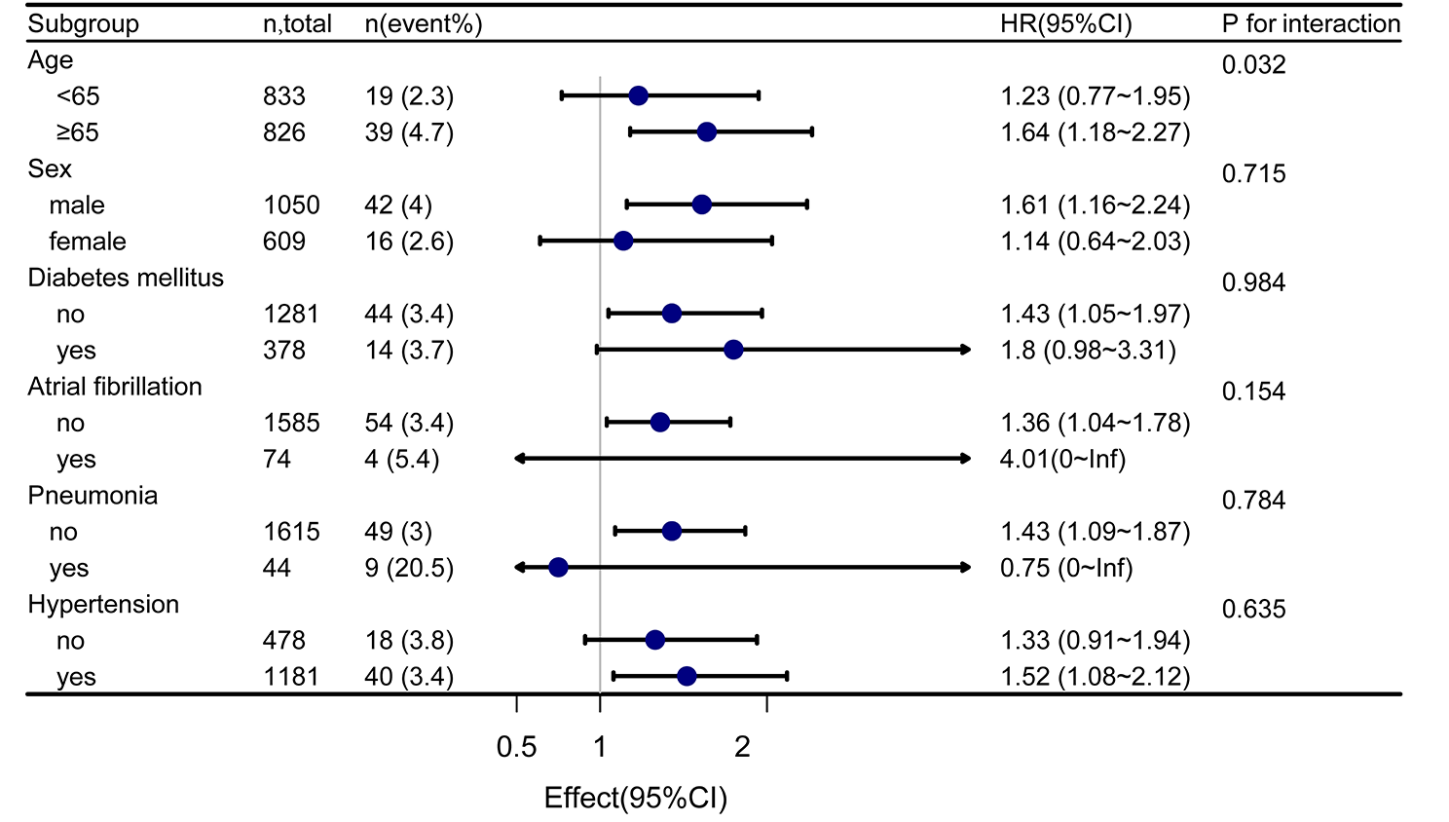
Forest map subgroup analysis of non-HDL-C/HDL-C ratio and 1-year risk of recurrent stroke. Stratified by age, sex, diabetes mellitus, atrial fibrillation, pneumonia, and hypertension

Since stroke patients often have complications that affect the patient’s prognosis, we performed subgroup and interactive analyses to assess whether the relationship between the non-HDL-C/HDL-C ratio and the 1-year risk of recurrent stroke was consistent in different subgroups (Fig. 4). The results indicated a significant increase in the 1-year risk of recurrent stroke in NICE patient aged ≥ 65 years with an increase in the non-HDL-C/HDL-C ratio. Furthermore, a significant interaction was observed between patients aged < 65 years and those aged ≥ 65 years (interaction p = 0.032). Although subgroup analysis showed no significant interaction effect in different sexes (*P* for interaction = 0.715), the 1-year risk of recurrent stroke in men (HR: 1.61, 95%CI: 1.16–2.24) was higher than that in women. Similar results were found in NICE patients without diabetes mellitus (HR: 1.43, 95%CI: 1.05–1.97), without atrial fibrillation (HR: 1.36, 95%CI: 1.04–1.78), and with hypertension (HR: 1.52, 95%CI: 1.08–2.12). There was no significant difference in the interactive analyses.

## Discussion

Our study showed that the non-HDL-C/HDL-C ratio was an independent risk factor for recurrent stroke within 1 year in patients with NICE. A significant increase in the 1-year risk of recurrent stroke caused by increased non-HDL-C/HDL-C ratio was mainly observed in older patients with NICE but not those aged < 65 years. In the subgroup analysis, the 1-year risk of recurrent stroke increased with an elevated non-HDL-C/HDL-C ratio for men and patients with hypertension, but it was not statistically significant in other comorbidities.

Mounting evidence indicates that nontraditional lipid indicators are associated with stroke prognosis [5, 6]. However, few studies have focused on the relationship between the non-HDL-C/HDL-C ratio and the risk of recurrent stroke, especially in patients with NICE. In this study, our findings indicate that the increased 1-year risk of recurrent stroke was statistically significant only in the adjusted model but not in the crude model (Table 2). After additional covariate screening and stratified analysis, our results demonstrate that age is a crucial factor in the relationship between the non-HDL-C/HDL-C ratio and the 1-year risk of recurrent stroke, with 65 years of age identified as the optimal cut-off point (Supplementary Table 2). Specifically, there was a significant increase in the 1-year risk of recurrent stroke associated with an increased non-HDL-C/HDL-C ratio in older patients with NICE, while no such association was observed in those aged < 65 years (Table 3). This result is similar to that of Guo et al.’s, which found that the non-HDL-C/HDL-C ratio as a coefficient of atherosclerosis was associated with intracranial arterial stenosis, a potential risk factor for recurrent stroke [7]. This finding suggests that clinicians should pay attention to the non-HDL-C/HDL-C ratio in older patients with NICE and that early interventions may help reduce the 1-year risk of recurrent stroke.

To explain why the non-HDL-C/HDL-C ratio only significantly increased the 1-year risk of recurrent stroke in age ≥65 years patients with NICE, we conducted a thorough literature review and comparative analysis. The possible explanations are as follows: First, previous studies have shown that the non-HDL-C/HDL-C ratio was significantly associated with an increased prevalence of intracranial arterial stenosis, leading to an increased degree of luminal stenosis, plaque lipid area, intra-plaque hemorrhage, and thrombosis [7, 9, 22, 23].These are independent risk factors for recurrent stroke. Older patients are more susceptible to the effects of the non-HDL-C/HDL-C ratio due to age-related changes in immunity, organ function, blood vessels elasticity, and blood flow velocity, which increase the risk of thrombosis. Second, the proportion of ever smokers was similar across the four groups (Q1-Q4), but the smoking cessation rate was highest in Q1, suggesting that the higher non-HDL/HDL ratio in younger patients may be a reflection of poor health behavior in younger patients. In contrast, older patients in Q4 may have a high non-HDL/HDL ratio despite adequate medical treatment including statin prescription and thus have a higher “true” atherosclerotic burden at baseline. This may be one of the reasons why this indicator only significantly affects stroke recurrence in older patients. Third, some studies have revealed that an elevated non-HDL-C/HDL-C ratio can lead to the occurrence and development of chronic kidney disease and metabolic syndrome [12, 24–26]. Compared with NICE patients aged < 65 years, older patients had lower eGFR levels and higher serum creatinine and blood urea nitrogen levels, suggesting that older patients with NICE are at risk of chronic renal impairment, thus increasing the risk of recurrent stroke. In addition, our results showed that HDL cholesterol, TG levels, and SBP on admission were significantly higher in older patients with NICE than in those aged < 65 years (Supplementary Table 3). Per the criteria for metabolic syndrome [27], the metabolic syndrome risk is significantly increased in older patients with NICE [28]. Fourth, related studies have shown that high non-HDL-C and low HDL-C levels were independent risk factors for mild cognitive impairment [29, 30], more common in older patients with stroke, and associated with atrophy of the hippocampus and recurrent stroke [31–34]. One study showed that approximately 30% of older patients with mild cognitive impairment experience recurrent strokes [33]. These findings suggest that an elevated non-HDL-C/HDL-C ratio (non-HDL-C divided by HDL-C levels) may lead to an increased risk of recurrent stroke by influencing cognitive impairment.

In addition to the above possible explanations, our comparative analysis also found that older patients with NICE have a higher proportion of elementary education background or below, higher NIHSS score on admission, and a significantly higher proportion of prior stroke, pneumonia, and atrial fibrillation than those < 65 years (Supplementary Table 3). These clinical characteristics suggest that, although NICE patients have mild symptoms, older patients with NICE also have more comorbidities associated with an increased risk of recurrent stroke.

To analyze the effect of comorbidities in patients with NICE on our results, we performed a subgroup analysis to explore this issue. There was no statistically significant interaction between the non-HDL-C/HDL-C ratio and the 1-year risk of recurrent stroke in each subgroup. However, the subgroup analysis showed a higher 1-year risk of recurrent stroke in men than in women with NICE (Fig. 4). This finding may be related to the higher proportion of male patients engaged in smoking and drinking and experiencing chronic renal injury (Supplementary Table 4). This sex difference is similar to the results of a previous study that found male patients with high internal carotid artery stenosis had an increased risk of recurrent stroke, mainly due to the high proportion of smoking and high serum creatinine and uric acid levels [35]. Subgroup analysis showed that patients with NICE with hypertension had a higher 1-year risk of recurrent stroke with an elevated non-HDL-C/HDL-C ratio than patients without hypertension. This result is similar to previous studies that found a history of hypertension and elevated SBP after the initial stroke was associated with an increased risk of recurrent stroke [36–38]. Our results also showed that the 1-year risk of recurrent stroke was increased in patients with NICE without diabetes mellitus and atrial fibrillation. However, there are no relevant reports on this aspect. Further analysis of the effect of comorbidities in patients with NICE on the relationship between non-HDL-C/HDL-C ratio and recurrent stroke within 1 year is needed.

Our study has some limitations. First, we only included patients with stroke from four tertiary grade A hospitals in Xi’an, China, which could not represent the status quo of patients with NICE in community hospitals. Second, the imaging indicators related to patients with NICE were not included in our study. Therefore, we did not adjust the indicators, which may have a particular effect on the results. Third, our study did not include the indicators related to the neck, peripheral blood, and intracranial vessels. Finally, since previous studies have not focused on the relationship between the non-HDL-C/HDL-C ratio and recurrent stroke in patients with NICE, our results need to be further verified by subsequent studies.

## Conclusions

Our study found that an elevated non-HDL-C/HDL-C ratio significantly increased the 1-year risk of recurrent stroke in older patients with NICE but not in those aged < 65 years. Age was an interaction factor between the non-HDL-C/HDL-C ratio and recurrent stroke. The optimal range of the non-HDL-C/HDL-C ratio should be no higher than Q2 (2.256–2.939). Our results suggest that clinicians assessing older patients with NICE with an appropriate range of non-HDL-C/HDL-C ratios may help reduce the 1-year risk of recurrent stroke.

## Electronic supplementary material

Below is the link to the electronic supplementary material.

Supplementary Material 1

Supplementary Material 2

## Data Availability

The data that support the findings of this study are available on request from the corresponding author. The data are not publicly available due to privacy or ethical restrictions.

## List of abbreviations

CI: confidence interval
BMI: body mass index
DBP: diastolic blood pressure
FBG: fasting blood glucose
eGFR: estimated glomerular filtration rate
HDL-C: high-density lipoprotein cholesterol
HR: hazard ratio
LDL-C: low-density lipoprotein cholesterol
NICE: non-disabling ischemic cerebrovascular events
NIHSS: National Institute of Health Stroke Scale
non-HDL-C: non-high-density lipoprotein cholesterol
SBP: systolic blood pressure
TC: total cholesterol
TGs: triglycerides
TIA: transient ischemic attack
