# Supplementary material for: Elevated non-HDL-C/HDL-C ratio increases the 1-year risk of recurrent stroke in older patients with non-disabling ischemic cerebrovascular events: results from the Xi’an Stroke Registry Study of China
Source: BMC Geriatr. 2023 Jul 5;23:410. doi: 10.1186/s12877-023-04102-x (PMC10320883; doi:10.1186/s12877-023-04102-x)
Supplement: Supplementary file 1 — Supplementary Material 1 [file 12877_2023_4102_MOESM1_ESM.docx]

**Supplementary table 1 Differences in** **clinical characteristics of the study group were compared with those of the lost follow-up group**

| Variables | Overall  n=1854 | Not loss to follow-up  at 1 year, n=1659 | Loss to follow-up  at 1 year, n=195 | *P*-value |
| --- | --- | --- | --- | --- |
| **Demographic information** |  |  |  |  |
| Age (years) | 64.0±12.2 | 64.0±12.1 | 63.7±13.1 | 0.687 |
| Sex, n(%) |  |  |  | 0.396 |
| male | 1180(63.6) | 1050(63.3) | 130(66.7) |  |
| female | 674(36.4) | 609(36.7) | 65(33.3) |  |
| Educational level, n (%) |  |  |  | 0.058 |
| elementary or below | 834(45.0) | 756(45.6) | 78(40) |  |
| middle school | 351(18.9) | 302(18.2) | 49(25.1) |  |
| high school or above | 669(36.1) | 601(36.2) | 68(34.9) |  |
| **Cerebrovascular risk factors** |  |  |  |  |
| Smoking, n (%) |  |  |  | 0.362 |
| Never smoking | 1032(55.7) | 925(55.8) | 107(54.9) |  |
| smoking cessation | 363(19.6) | 318(19.2) | 45(23.1) |  |
| current smoking | 459(24.8) | 416(25.1) | 43(22.1) |  |
| Drinking, n (%) | 453(24.4) | 400(24.1) | 53(27.2) | 0.392 |
| **Examination on admission** |  |  |  |  |
| BMI (kg/m^2^) | 24.0±3.3 | 24.0±3.3 | 23.9±2.9 | 0.865 |
| SBP on admission (mmHg) | 145.0±20.7 | 145.4±20.8 | 141.9±19.9 | 0.029 |
| DBP on admission (mmHg) | 85.3±12.1 | 85.5±12.3 | 83.7±10.6 | 0.048 |
| Heart rate (times per minute) | 74.5±9.7 | 74.5±9.6 | 75.1±10.0 | 0.352 |
| NIHSS score on admission,(IQR) | 2.0(1.0,4.0) | 2.0(1.0,4.0) | 2.0(1.0,4.0) | 0.668 |
| **Previous medical history** |  |  |  |  |
| Prior stroke, n (%) | 492(26.5) | 442(26.6) | 50(25.6) | 0.831 |
| Pneumonia, n (%) | 46(2.5) | 44(2.7) | 2(1) | 0.224 |
| Hypertension, n (%) | 1311(70.7) | 1181(71.2) | 130(66.7) | 0.219 |
| Diabetes mellitus, n (%) | 427(23.0) | 378(22.8) | 49(25.1) | 0.519 |
| Atrial fibrillation, n (%) | 80(4.3) | 74(4.5) | 6(3.1) | 0.476 |
| **Laboratory findings** |  |  |  |  |
| Total cholesterol (mmol/L) | 4.4±1.0 | 4.4±1.1 | 4.4±0.9 | 0.591 |
| Triglycerides (mmol/L) | 1.7±1.3 | 1.7±1.4 | 1.6±1.2 | 0.326 |
| HDL-cholesterol (mmol/L) | 1.1±0.3 | 1.1±0.3 | 1.2±0.3 | 0.179 |
| LDL-cholesterol (mmol/L) | 2.6±0.8 | 2.6±0.8 | 2.6±0.8 | 0.926 |
| Gycosylated hemoglobin,% | 6.4±1.6 | 6.4±1.6 | 6.5±1.8 | 0.399 |
| FBG (mmol/L) | 5.9±2.3 | 5.9±2.3 | 5.8±2.5 | 0.66 |
| Alanine aminotransferase (U/L) | 23.5±18.2 | 23.7±18.4 | 21.9±15.7 | 0.182 |
| Aspartate aminotransferase (U/L) | 23.8±12.7 | 23.8±12.5 | 23.3±13.6 | 0.599 |
| Alkaline phosphatase (U/L) | 78.4±25.7 | 78.3±25.4 | 79.7±27.9 | 0.481 |
| Homocysteine (μmol/mL) | 21.1±14.2 | 21.1±14.2 | 21.2±14.3 | 0.928 |
| Serum [creatinine](javascript:;) (μmol/L) | 75.6±37.0 | 75.8±38.5 | 73.9±21.7 | 0.507 |
| eGFR (mL/min/1.73m^2^) | 75.7±17.8 | 75.8±17.7 | 75.3±18.5 | 0.71 |
| Blood Urea Nitrogen (mmol/L) | 5.0±1.8 | 5.0±1.8 | 5.1±1.8 | 0.635 |
| Uric acid (μmol/L) | 293.2±96.4 | 292.6±96.5 | 298.0±96.2 | 0.463 |
| Leukocyte count (×10^9^/L) | 6.7±2.3 | 6.7±2.3 | 6.6±2.3 | 0.696 |
| Platelet count (×10^9^/L) | 191.3±59.2 | 191.0±59.2 | 193.8±59.6 | 0.535 |

Abbreviations: BMI, body mass index; FBG, fasting blood glucose; NIHSS, National Institutes of Health Stroke Scale; SBP, systolic blood pressure; DBP, diastolic blood pressure; HDL, high-density lipoprotein; LDL, low-density lipoprotein; eGFR, estimated glomerular filtration rate.

**Supplementary table 2: Cox regression analysis of the non-HDL-C/HDL-C ratio and recurrent stroke within 1 year stratified by age.**

| Stratified by age | n, total | n (%) | Crude model  HR(95CI) | *P* value | Adjusted model  HR(95CI) | *P* value |
| --- | --- | --- | --- | --- | --- | --- |
| <55 years | 358 | 7(2) | 0.87(0.46~1.63) | 0.66 | Inf (0~inf) | 0.299 |
| 55-64 years | 475 | 12(2.5) | 0.93(0.59~1.47) | 0.765 | 1.25(0.66~2.34) | 0.494 |
| 65-74 years | 456 | 17(3.7) | 1.53(1.06~2.22) | 0.024 | 1.92(1.16~3.18) | 0.011 |
| ≥75 years | 370 | 22(5.9) | 1.25(0.84~1.85) | 0.275 | 1.73(1.04~2.88) | 0.036 |

Notes: Crude adjust none; adjusted for age, sex; smoking, drinking, prior stroke, pneumonia, NIHSS score at admission, BMI, ALT, triglyceride, FBG, alkaline phosphatase, platelet count, blood urea nitrogen, hypertension, atrial fibrillation, and diabetes mellitus. Abbreviations: BMI, body mass index; FBG, fasting blood glucose; NIHSS, national institutes of health stroke scale; ALT, alanine aminotransferase; HR, hazard ratio; CI, confidence interval;

**Supplementary table 3 Comparison of clinical characteristics between age<65 year and age ≥ 65 year**

| Variables | Overall  n=1659 | Age<65 year,  n=833 | Age≥65 year,  n=826 | *P*-value |
| --- | --- | --- | --- | --- |
| **Demographic information** |  |  |  |  |
| Age (years) | 64.0±12.1 | 54.2±7.9 | 73.9±6.0 | <0.001 |
| Sex, n(%) |  |  |  | <0.001 |
| male | 1050(63.3) | 576(69.1) | 474(57.4) |  |
| female | 609(36.7) | 257(30.9) | 352(42.6) |  |
| Educational level, n (%) |  |  |  | <0.001 |
| elementary or below | 756(45.6) | 328(39.4) | 428(51.8) |  |
| middle school | 302(18.2) | 186(22.3) | 116(14) |  |
| high school or above | 601(36.2) | 319(38.3) | 282(34.1) |  |
| **Cerebrovascular risk factors** |  |  |  |  |
| Smoking, n (%) |  |  |  | <0.001 |
| Never smoking | 925(55.8) | 404(48.5) | 521(63.1) |  |
| smoking cessation | 318(19.2) | 146(17.5) | 172(20.8) |  |
| current smoking | 416(25.1) | 283(34) | 133(16.1) |  |
| Drinking, n (%) | 400(24.1) | 264(31.7) | 136(16.5) | <0.001 |
| **Examination on admission** |  |  |  |  |
| BMI (kg/m^2^) | 24.0±3.3 | 24.4±3.3 | 23.5±3.3 | <0.001 |
| SBP on admission (mmHg) | 145.4±20.8 | 143.9±20.9 | 146.9±20.5 | 0.004 |
| DBP on admission (mmHg) | 85.5±12.3 | 87.8±12.7 | 83.2±11.3 | <0.001 |
| Heart rate (times per minute) | 74.5±9.6 | 74.6±8.9 | 74.4±10.3 | 0.671 |
| NIHSS score on admission,(IQR) | 2.0(1.0,4.0) | 2.0(0.0,4.0) | 3.0(1.0,4.0) | 0.023 |
| **Previous medical history** |  |  |  |  |
| prior stroke, n (%) | 442(26.6) | 191(22.9) | 251(30.4) | <0.001 |
| Pneumonia, n (%) | 44(2.7) | 12(1.4) | 32(3.9) | 0.003 |
| Hypertension, n (%) | 1181(71.2) | 573(68.8) | 608(73.6) | 0.035 |
| Diabetes mellitus, n (%) | 378(22.8) | 195(23.4) | 183(22.2) | 0.582 |
| Atrial fibrillation, n (%) | 74(4.5) | 21(2.5) | 53(6.4) | <0.001 |
| **Laboratory findings** | |  |  |  |
| Total cholesterol (mmol/L) | 4.4±1.1 | 4.4±1.1 | 4.4±1.0 | 0.07 |
| Triglycerides (mmol/L) | 1.4(1.0,2.0) | 1.5(1.1,2.2) | 1.3(1.0,1.8) | <0.001 |
| HDL-cholesterol (mmol/L) | 1.1±0.3 | 1.1±0.3 | 1.2±0.3 | <0.001 |
| LDL-cholesterol (mmol/L) | 2.6±0.8 | 2.6±0.9 | 2.6±0.8 | 0.232 |
| Gycosylated hemoglobin,% | 6.4±1.6 | 6.4±1.6 | 6.3±1.5 | 0.657 |
| FBG (mmol/L) | 5.9±2.3 | 6.0±2.4 | 5.7±2.1 | 0.025 |
| Alanine aminotransferase (U/L) | 19.0(14.0,27.4) | 21.8(16.0,31.0) | 17.0(13.0,24.0) | <0.001 |
| Aspartate aminotransferase (U/L) | 21.0(17.0,27.0) | 21.0(17.0,28.0) | 20.6(17.0,26.0) | 0.096 |
| Alkaline phosphatase (U/L) | 78.3±25.4 | 78.7±24.2 | 77.9±26.6 | 0.537 |
| Homocysteine (μmol/mL) | 21.1±14.2 | 21.4±15.1 | 20.7±13.2 | 0.442 |
| Serum [creatinine](javascript:;) (μmol/L) | 75.8±38.5 | 73.5±29.6 | 78.1±45.6 | 0.017 |
| eGFR (mL/min/1.73m^2^) | 75.8±17.7 | 80.6±17.6 | 70.9±16.5 | <0.001 |
| Blood Urea Nitrogen (mmol/L) | 5.0±1.8 | 4.8±1.6 | 5.3±2.0 | <0.001 |
| Uric acid (μmol/L) | 292.6±96.5 | 291.9±93.9 | 293.3±99.0 | 0.78 |
| Leukocyte count (×10^9^/L) | 6.7±2.3 | 6.8±2.1 | 6.6±2.4 | 0.015 |
| Platelet count (×10^9^/L) | 191.0±59.2 | 196.7±59.0 | 185.3±58.9 | <0.001 |

Abbreviations: BMI, body mass index; FBG, fasting blood glucose; NIHSS, National Institutes of Health Stroke Scale; SBP, systolic blood pressure; DBP, diastolic blood pressure; HDL, high-density lipoprotein; LDL, low-density lipoprotein; eGFR, estimated glomerular filtration rate.

**Supplementary table 4 Comparison of clinical characteristics between male and female**

| Variables | Overall  n=1659 | Male,  n=1050 | Female,  n=609 | *P* value |
| --- | --- | --- | --- | --- |
| **Demographic information** |  |  |  |  |
| Age (years) | 64.0±12.1 | 62.7±12.2 | 66.2±11.6 | <0.001 |
| Educational level, n (%) |  |  |  | <0.001 |
| elementary or below | 756(45.6) | 418(39.8) | 338(55.5) |  |
| middle school | 302(18.2) | 205(19.5) | 97(15.9) |  |
| high school or above | 601(36.2) | 427(40.7) | 174(28.6) |  |
| **Cerebrovascular risk factors** |  |  |  |  |
| Smoking, n (%) |  |  |  | <0.001 |
| Never smoking | 925(55.8) | 327(31.1) | 598(98.2) |  |
| smoking cessation | 318(19.2) | 314(29.9) | 4(0.7) |  |
| current smoking | 416(25.1) | 409(39) | 7(1.1) |  |
| Drinking, n (%) | 400(24.1) | 394(37.5) | 6(1) | <0.001 |
| **Examination on admission** |  |  |  |  |
| BMI (kg/m^2^) | 24.0±3.3 | 24.0±3.4 | 23.9±3.2 | 0.383 |
| SBP on admission (mmHg) | 145.4±20.8 | 145.1±20.2 | 145.8±21.7 | 0.497 |
| DBP on admission (mmHg) | 85.5±12.3 | 85.8±12.4 | 85.0±12.0 | 0.179 |
| Heart rate (times per minute) | 74.5±9.6 | 74.0±9.3 | 75.3±10.2 | 0.005 |
| NIHSS score on admission,(IQR) | 2 (1, 4) | 3 (1, 4) | 2 (0, 4) | 0.029 |
| **Previous medical history** |  |  |  |  |
| prior stroke, n (%) | 442(26.6) | 295(28.1) | 147(24.1) | 0.089 |
| Pneumonia, n (%) | 44(2.7) | 32(3) | 12(2) | 0.247 |
| Hypertension, n (%) | 1181(71.2) | 726(69.1) | 455(74.7) | 0.018 |
| Diabetes mellitus, n (%) | 378(22.8) | 234(22.3) | 144(23.6) | 0.565 |
| Atrial fibrillation, n (%) | 74(4.5) | 46(4.4) | 28(4.6) | 0.934 |
| **Laboratory findings** |  |  |  |  |
| Total cholesterol (mmol/L) | 4.4±1.1 | 4.2±1.0 | 4.7±1.1 | <0.001 |
| Triglycerides (mmol/L) | 1.7±1.4 | 1.7±1.4 | 1.8±1.2 | 0.458 |
| HDL-cholesterol (mmol/L) | 1.1±0.3 | 1.1±0.3 | 1.2±0.3 | <0.001 |
| LDL-cholesterol (mmol/L) | 2.6±0.8 | 2.5±0.8 | 2.8±0.9 | <0.001 |
| Non-HDL-cholesterol (mmol/L) | 3.3±1.0 | 3.1±0.9 | 3.5±1.0 | <0.001 |
| Non-HDL-c/HDL-c | 3.1±1.2 | 3.1±1.2 | 3.0±1.1 | 0.198 |
| Glycosylated hemoglobin,% | 6.4±1.6 | 6.3±1.5 | 6.5±1.7 | 0.035 |
| FBG (mmol/L) | 5.9±2.3 | 5.8±2.2 | 6.0±2.3 | 0.012 |
| Alanine aminotransferase (U/L) | 19.0(14.0,27.4) | 20.0(14.8,28.3) | 18.0(13.0,26.0) | <0.001 |
| Aspartate aminotransferase (U/L) | 23.8±12.5 | 23.8±12.0 | 23.8±13.4 | 0.936 |
| Alkaline phosphatase (U/L) | 78.3±25.4 | 76.8±24.3 | 80.9±27.1 | 0.002 |
| Homocysteine (μmol/mL) | 21.1±14.2 | 23.1±15.3 | 17.7±11.3 | <0.001 |
| Serum [creatinine](javascript:;) (μmol/L) | 75.8±38.5 | 81.4±42.7 | 66.0±27.0 | <0.001 |
| eGFR (mL/min/1.73m^2^) | 75.8±17.7 | 74.5±16.6 | 78.0±19.4 | <0.001 |
| Blood Urea Nitrogen (mmol/L) | 5.0±1.8 | 5.2±1.8 | 4.8±1.8 | <0.001 |
| Uric acid (μmol/L) | 292.6±96.5 | 309.2±97.8 | 263.4±86.8 | <0.001 |
| Leukocyte count (×10^9^/L) | 6.7±2.3 | 6.8±2.2 | 6.5±2.4 | 0.013 |
| Platelet count (×10^9^/L) | 191.0±59.2 | 184.8±57.3 | 201.8±60.9 | <0.001 |

Abbreviations: BMI, body mass index; FBG, fasting blood glucose; NIHSS, National Institutes of Health Stroke Scale; SBP, systolic blood pressure; DBP, diastolic blood pressure; HDL, high-density lipoprotein; LDL, low-density lipoprotein; eGFR, estimated glomerular filtration rate.
